# Management and prognosis of a vital cracked tooth by occlusal veneer for 14 months: A case report

**DOI:** 10.1002/ccr3.7714

**Published:** 2023-08-08

**Authors:** Mengke Wang, Yingying Hong, Xiaomei Hou, Yinfei Pu

**Affiliations:** ^1^ Second Clinical Division Peking University School and Hospital of Stomatology and National Center for Stomatology and National Clinical Research Center for Oral Diseases and National Engineering Research Center of Oral Biomaterials and Digital Medical Devices Beijing People's Republic of China; ^2^ First Clinical Division Peking University School and Hospital of Stomatology and National Center for Stomatology and National Clinical Research Center for Oral Diseases and National Engineering Research Center of Oral Biomaterials and Digital Medical Devices Beijing People's Republic of China; ^3^ Department of Oral Emergency Peking University School and Hospital of Stomatology and National Center for Stomatology and National Clinical Research Center for Oral Diseases and National Engineering Research Center of Oral Biomaterials and Digital Medical Devices Beijing People's Republic of China

**Keywords:** cracked tooth, minimally invasive dentistry, occlusal veneer, vital cracked tooth

## Abstract

**Key Clinical Message:**

An occlusal veneer is an ultrathin restoration method and a minimally invasive approach that can preserve more dental tissue and provide better aesthetic outcomes, thus increasing patient satisfaction.

**Abstract:**

An occlusal veneer is an ultrathin restoration method and a minimally invasive approach that can preserve more dental tissue and provide better aesthetic outcomes, thus increasing patient satisfaction; however, no previous studies reported on treating cracked teeth using occlusal veneer. Accordingly, we described the diagnosis and treatment process of a cracked tooth using occlusal veneer in a single case. A 29‐year‐old male presented at our dental clinic complaining of biting pain in the mandibular molar on the right‐hand side. A routine oral examination with radiography was performed to evaluate the oral condition and treatment planning. The #16 tooth had a crack line surrounding the whole distal–lingual cusp from the occlusal surface. After discussing various therapeutic options with the patient, an occlusal veneer was performed. One week after treatment with occlusal veneer, the patient had no complaints. A 14‐month follow‐up showed promising clinical and radiographic outcomes. Occlusal veneer is an alternative treatment option for a cracked tooth, as it can preserve more dental tissue and potentially save pulp vitality.

## Graphical abstract

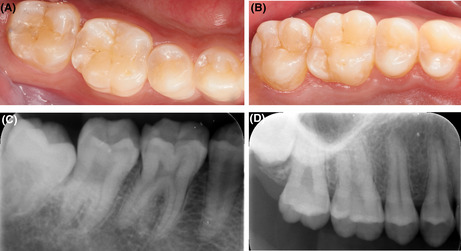

## 1 INTRODUCTION

Cracked tooth syndrome, also known as a fractured tooth, is a common, well‐documented condition in children and older people. It is often small and harmless and occurs due to morphologic, physical, and iatrogenic factors, such as steep cusp to fossa inclination, bruxism, clenching, extensive attrition, or abrasion.^1^ A cracked tooth is defined as a thin surface disruption of enamel and dentin of unknown depth or extension or an incomplete fracture initiated from the crown extending subgingivally, usually directed mesiodistally.^2^ Various clinical symptoms have been reported depending on the direction of the crack line and the extension of the crack.^3^ Common symptoms include pain on release after biting, pain on biting, and thermal sensitivity.^4^ Nevertheless, the pain from a cracked tooth can be difficult to distinguish from other conditions, such as atypical orofacial or ear pain, migraine, temporomandibular joint disorders, and sinusitis. Thus, establishing a diagnosis may be clinically challenging.^5^ However, even with the assistance of various diagnostic methods, the diagnosis is also difficult because diverse factors cause it, and the symptoms vary depending on the severity of the crack. Therefore, early detection and appropriate treatment are important for retaining the tooth.

Managing cracked teeth can also be challenging, as it involves mechanical and biological considerations. The treatment often consists of root canal therapy (RCT) according to the pulpal and periradicular diagnosis and full cuspal coverage with bonded restorative materials. Full cuspal coverage restorations (i.e., full crown) have been used for many years to restore esthetics, occlusion, and function, showing satisfactory clinical and mechanical performance.^6^ Mechanically, cuspal coverage can splint the incompletely fractured segments, reducing cusp flexure.^7^ Still, for the vital cracked teeth treated with a full crown, loss of pulp vitality is the most frequently reported biological complication, with an incidence rate of 2.1% at 5 years, which can reach as high as 15%.^6^

An occlusal veneer is an ultrathin restoration that relies on bonding for retention and can completely cover cusps. It is used to treat occlusal developmental defects, chemical or mechanical abrasion, and reconstruct some occlusal surface morphology.^8^ Compared to a full crown, an occlusal veneer is less invasive, can preserve dental tissue, and provides better aesthetic outcomes and higher patient satisfaction.^9^, ^10^, ^11^ Nevertheless, no previous studies reported on treating cracked teeth using occlusal veneer.

In this study, we described the diagnosis and treatment process in a single case of a cracked tooth using occlusal veneer.

## 2 CASE REPORT

### 2.1 Medical history and clinical findings

A 29‐year‐old male visited our department (2nd Dental Center, Peking University School and Hospital of Stomatology) and was referred for an endodontic consultation. The patient's primary complaint was pain associated with the mandibular molar on the right‐hand side when biting hard food. The patient had no significant medical history or systemic disease. In addition, he was avoiding chewing on the right side because of occasional sensitivity to cold (symptoms lasted for 4 months and were getting worse). The patient did not recall chewing recently on a hard object, nor did he have a parafunctional habit of bruxism and clenching. He also had no history of trauma or injury.

**FIGURE 1:**
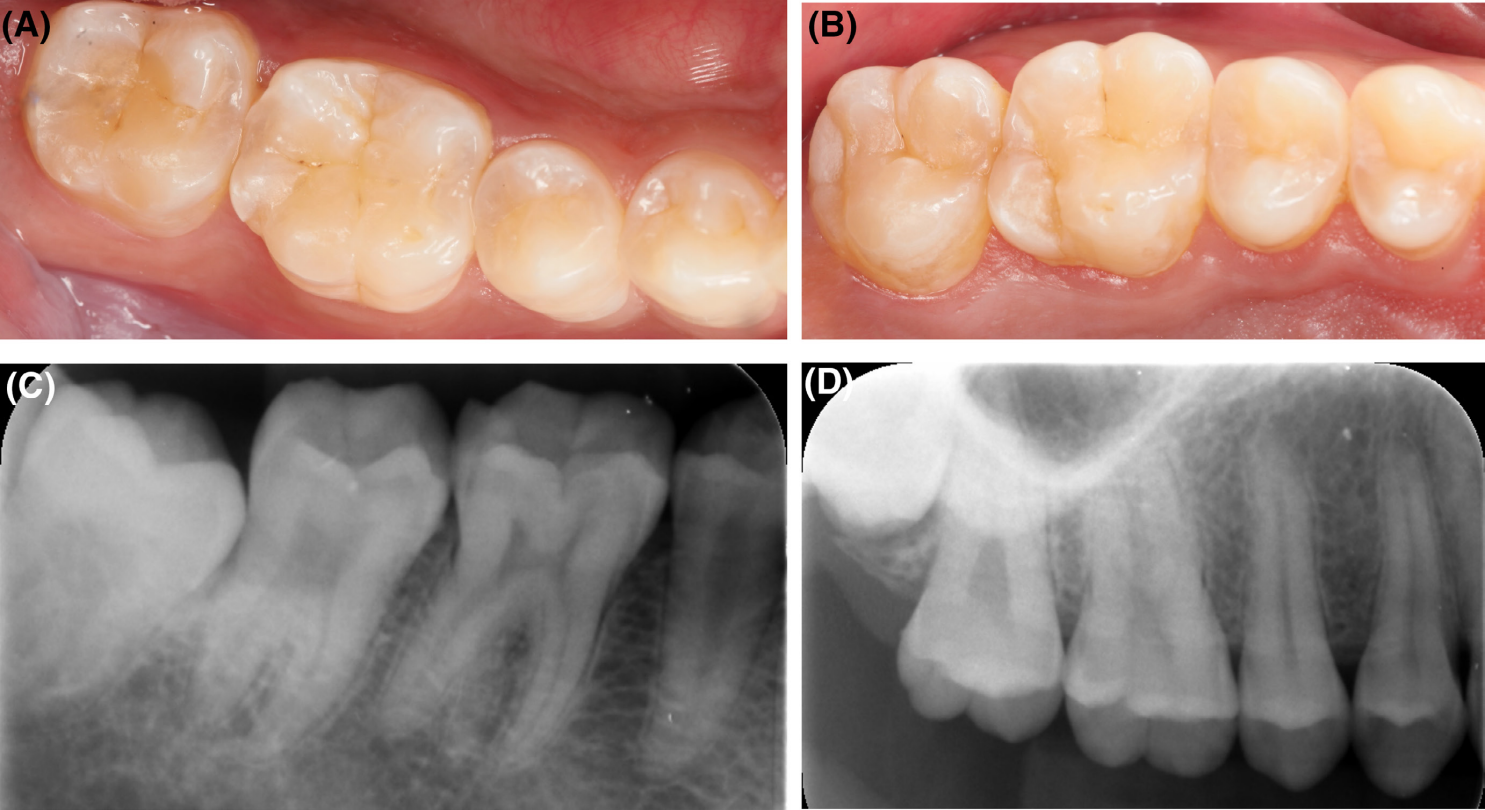
(A) Clinical presentation of the mandibular right molar region. All teeth were healthy and caries‐free, with age‐appropriate signs of attrition and without previous restorations. (B) Clinical presentation of the maxillary right molar region. The #16 tooth had a steep cusp with a crack line on the distal–lingual cusp. (C, D) The periapical radiograph of mandibular (C) and maxillary (D) right molar region. The X‐ray films revealed normal width of the periodontal ligament and without periapical lesion.

**FIGURE 2:**
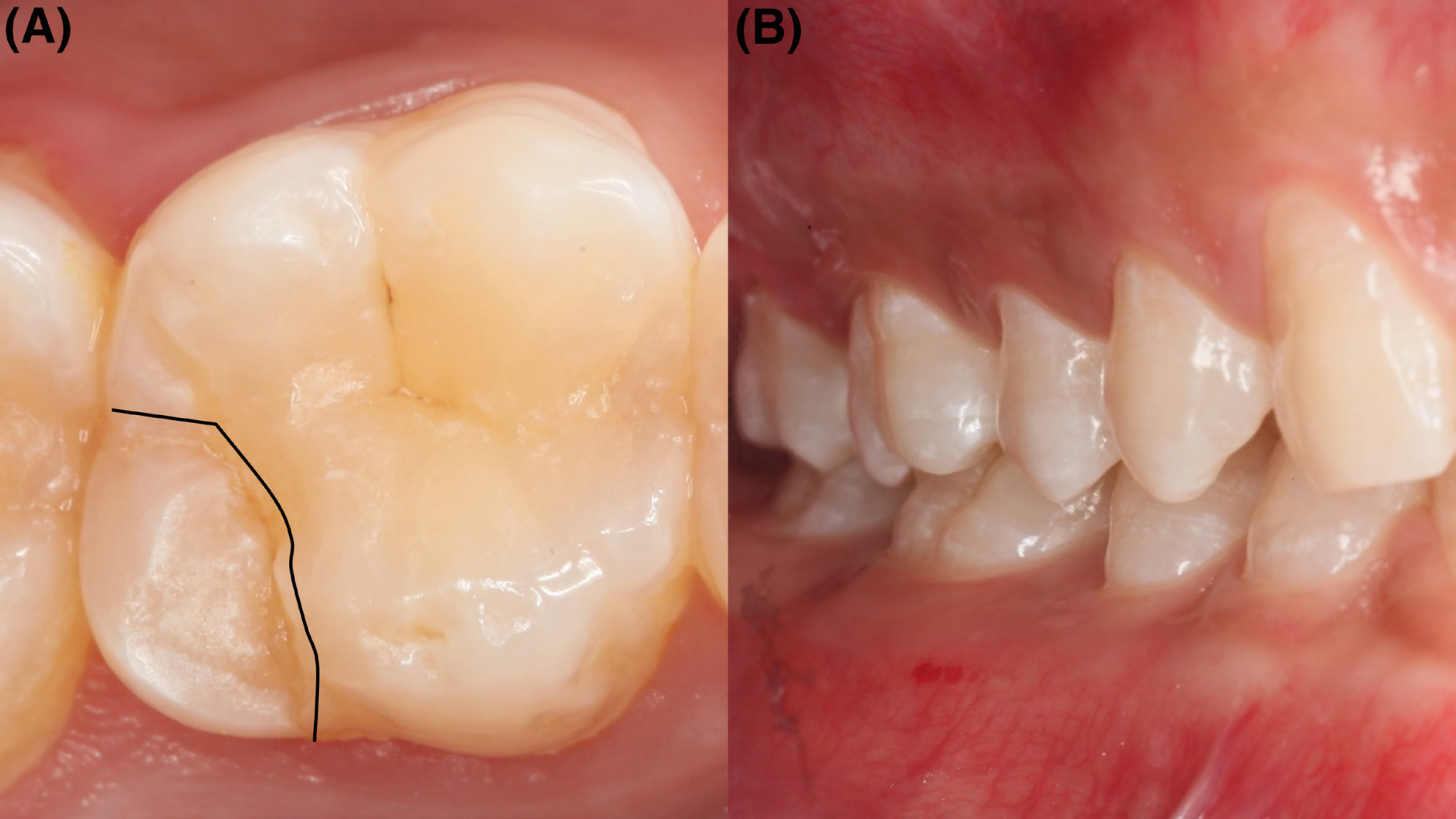
(A) The amplification clinical images of the #16 tooth displayed a distinct crack line on the distal–lingual cusp and surrounded the whole cusp from the occlusal surface as the black line displayed. (B) The clinical presentation of the lateral occlusal contacts in the right molar region. No occlusal interference and deep overbite and overjet were found.

Clinical and radiological examination (Figure 1) showed healthy teeth in the right mandibular region with no previous restorations or signs of periodontal disease. The final offending tooth was #16 with a steep cusp, which also had a distinct crack line on the distal–lingual cusp, involving the entire cusp from the occlusal surface (Figure 2).

### 2.2 Diagnosis

After confirming the offending tooth, detailed clinical and radiographic examinations were performed to obtain accurate pulpal and periapical diagnoses and periodontal and restorative assessment. As shown in Figure 2, the #16 tooth had a single crack line surrounding the distal–lingual cusp and no occlusal interference. Further intraoral examination revealed that the #16 tooth (Figure 1) was a healthy periodontium (with probing depth ≤3 mm), caries‐free tooth, without composite resin fillings, and with age‐appropriate signs of attrition.

A periapical radiograph in the distant paralleling technique of tooth #16 was taken, revealing a uniform periodontal ligament space associated with this tooth (Figure 1D). Pulp testing with refrigerant spray caused an immediate and short reaction (lasting 2, 3 s), suggesting normal pulp vitality. Moreover, the #16 cracked tooth exhibited no pain to percussion either from the vertical or the lateral direction. The offending tooth showed a biting sensitivity when examined with a small cotton ball. Tooth Slooth testing suggested that the distal–lingual cusp was symptomatic. Also, the cracked tooth had normal physical mobility and no palpation pain, spontaneous pain, abscess/swelling, or sinus tract.

After obtaining the patient's consent, the treatment plan was established. The advantages and risks of various therapeutic options, including direct restoration, the full crown, and the full crown with preventive RCT, were discussed with the patient. We recommended the minimally invasive therapeutic option (i.e., occlusal veneer) that included cuspal coverage restoration with less tooth preparation and did not require preventive RCT, which the patient eventually accepted.

### 2.3 Therapeutic strategy

**FIGURE 3:**
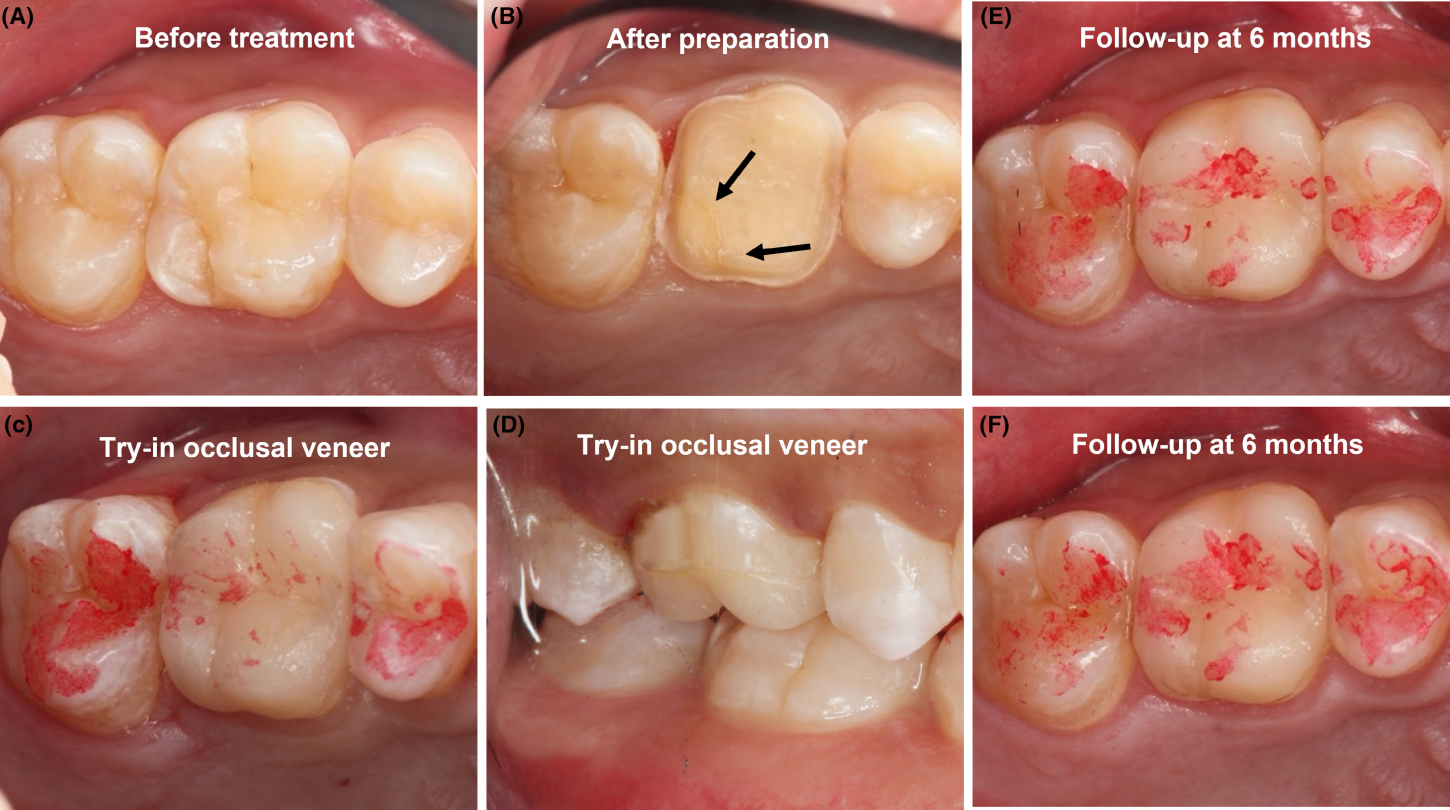
(A, B) The clinical presentation of the maxillary right first molar before treatment (A) and after intervention (B). The cracked line was distinct as the black arrow displayed. (C, D) The centric occlusal contact imprinting (C) and lateral occlusal contacts imprinting (D) after the occlusal veneer were try‐in repeatedly and bonded with the teeth. (E, F) The centric occlusal contact imprinting (E) and lateral occlusal contacts imprinting (F) after the occlusal veneer were used for 6 months.

The #16 cracked tooth was prepared as a standardized and cuspal‐covered occlusal veneer, with 0.5‐ to 1‐mm width rectangular or angular (slightly <90°) shoulder and a round and sharp inner line angle to fully cover the whole occlusal surface. In order to obtain effective pain control, local infiltration anesthesia with 4% articaine hydrochloride (containing epinephrine at a concentration of 1:100,000) was adopted before the operation. The functional cusps and the cusp with the crack line were reduced (dentin removed) by 1.0–1.5 mm parallel to the cusp incline with a coarse diamond rotary cutting instrument (Figure 3B).

As shown in Figure 3B, the orientation of the cracks was more apparent after the intervention was completed. The depth on the occlusal surface was 1.0–1.5 mm in thickness along the axial walls, which was needed to finish in an infinity bevel and supragingival. After preparation, the whole tooth surface was covered by the Hybrid coat (Sun Medical Co., Ltd.) to relieve the dentin sensitivity and avoid pulp stimulation. Moreover, we maintained partial cracks and ensured restorative space and the inexistence of caries, which was supported by the reported literature.^12^ In the study, the authors argued that keeping partial cracks could guarantee enough space for restorations and prevent further crack progression.^12^

After 2 weeks with a temporary restoration, no signs or symptoms of inflammation (by subjective symptom and cold test) were seen, so the definitive occlusal veneer was placed following the instructions. Next, the definitive occlusal veneer was tried‐in repeatedly to fit the occlusal morphology, after which the final occlusal state at centric occlusal contact (Figure 3C) and lateral occlusal movement (Figure 3D) were recorded.

### 2.4 Data collection during follow‐up

Clinical follow‐up of the cracked tooth was reexamined at 1 week, 1, 2, 3, 6, and 12 months after surgery, and then annually after that. The presence or absence of symptoms, pulpal responses to the cold test, responses to palpation and percussion tests, and periodontal probing depths measurements were reassessed at each follow‐up. In addition, tooth #16 and both adjacent teeth were clinically tested for the painful reaction after loading the individual cusp using the Tooth Slooth. The success of the occlusal veneer was defined as the absence of signs or symptoms without progressive radiographic pathosis.

The biting pain was relieved after 1 week. The pulp testing with refrigerant spray was normal, and the tooth had a healthy periodontium with a probing depth ≤3 mm. Moreover, after applying the occlusal veneer, the tooth was asymptomatic with a percussion test and a biting test with a small cotton ball.

After 2 weeks, no signs or symptoms of inflammation were observed. The patient could bite in the mandibular right molar as in other tooth positions, and the pulp was normal with the control tooth.

**FIGURE 4:**
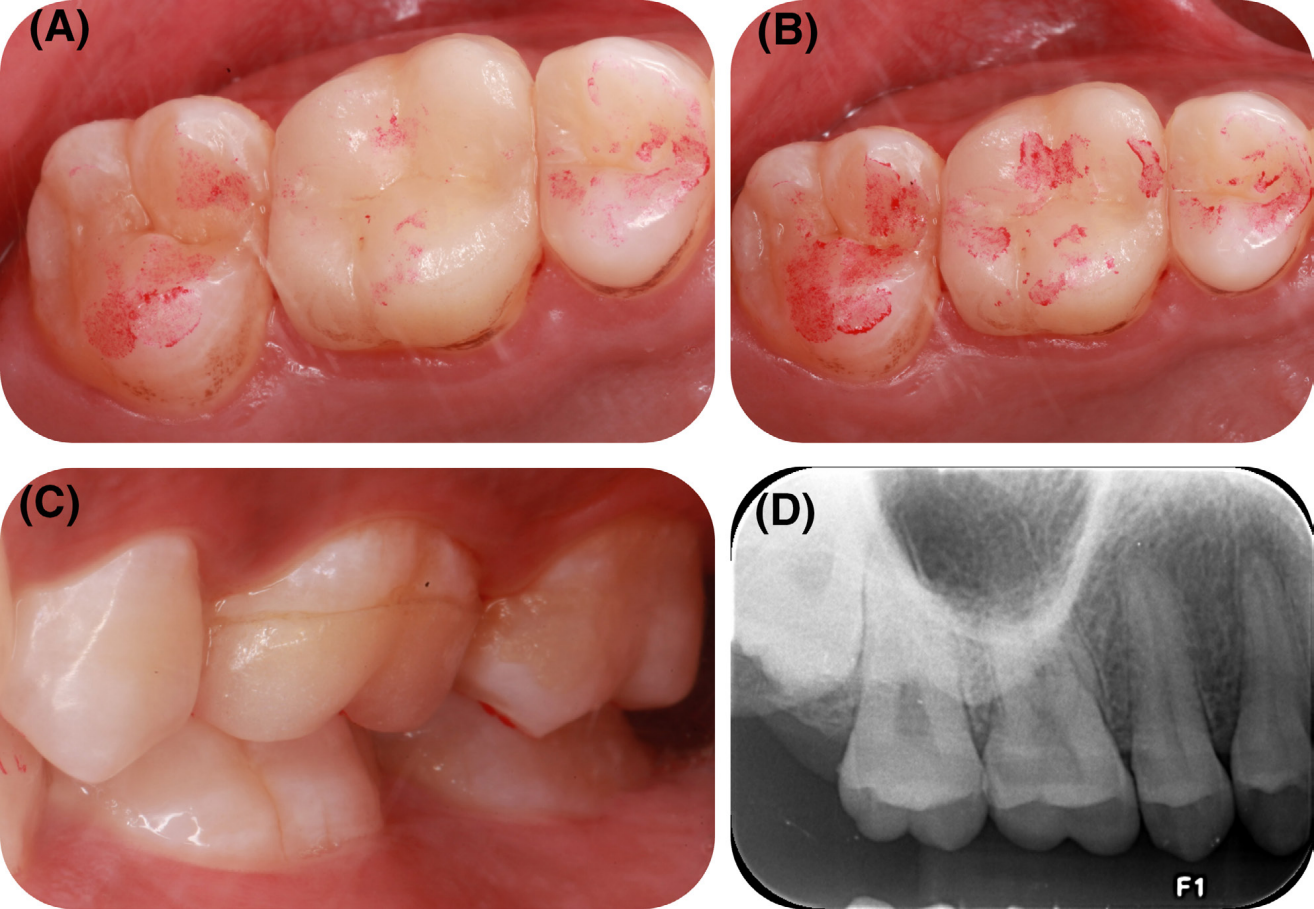
The clinical presentation of the maxillary right first molar after the occlusal veneer at 14 months follow‐up. The centric occlusal contact imprinting (A), and the centric and lateral occlusal contact imprinting (B) after intervention. The clinical presentation of lateral occlusal contacts (C) after the occlusal veneer at 14 months follow‐up. The periapical radiograph (D) of tooth #16 after the occlusal veneer at 14 months follow‐up. The X‐ray films revealed normal width of the periodontal ligament and without periapical lesion.

The occlusal examination (Figure 3E,F) at centric occlusal contact and lateral occlusal movement at 6 months showed a uniform and stable state of contact. The clinical occlusal examination at centric occlusal contact and lateral occlusal movement and radiological examination at 14 months are shown in Figure 4. After being treated with occlusal veneer for 14 months, the edge of the prosthesis was complete and continuous and had perfect marginal adaptation with the preparation. No occlusal interferences and parafunction were observed. A periapical radiograph of tooth #16 also revealed a uniform periodontal ligament space associated with this tooth.

## 3 DISCUSSION

Studies on managing a cracked tooth are rarely reported, making clinical decisions more difficult. Common symptoms of a cracked tooth include pain on release after biting, pain on biting, and thermal sensitivity.^4^ However, judging pain originating from Aδ and C fibers results in a subjective experience of a sharp and prickling pain, which is challenging to locate because of a high degree of convergence from pulp tissue and the lack of proprioceptive information provided.^13^ These results suggested that the teeth should be carefully examined to accurately identify the final offending tooth, especially when the cracks are indiscernible.

In the present study, the patient's primary complaint was pain associated with biting hard food in the mandibular right molar region. After performing a detailed clinical and radiological examination, a cracked tooth with vital pulp (#16 tooth) was located by visual inspection and biting pain. As previously reported, most patients can locate the offending teeth in the maxillary or mandible based on subjective symptoms, while diagnosis remains challenging for certain cases (these patients cannot identify the position of the tooth).^14^ Biting and thermal pain could be explained by the hydrodynamic theory, which is based on the concept that the rapid movement of dentinal fluid in the dentinal tubules stimulates mechanoreceptors near the odontoblast cell body and then activates the sensory nerve fibers in the dentin‐pulp complex.

Currently, cuspal coverage restoration is the cornerstone in managing cracked teeth.^15^ Cusp coverage, such as a full crown, removes the steep cuspal inclines, thus eradicating the phenomenon of wedge opening and decreasing the stress intensity factor at the crack tip. Yet, a 6‐year follow‐up study revealed that 20% of crowned cracked teeth with reversible pulpitis still require further root canal treatment.^12^ Vital teeth restored by the crown are also at a certain risk of applying RCT in the future because of pulp stimulus after preparation. Compared with a full coverage crown, the main advantages of occlusal veneer are simple preparation and no need to restrict clinical crown height because of bonding to retention.^9^, ^10^, ^11^ In our study, the patient's biting pain was relieved after 1 week of occlusal veneer restoration. Moreover, the #16 tooth kept a vital pulp state and was asymptomatic with percussion and biting test with a small cotton ball until follow‐up at 14 months. Yet, longer‐term follow‐up is needed to confirm the clinical and radiographic outcomes. Also, this tooth may still require root canal treatment in the future, as long‐term follow‐up (>5 years) regarding the management of cracked tooth syndrome via occlusal veneers has not yet been established in the literature.

## 4 CONCLUSION

The occlusal veneer is a feasible, effective, and minimally invasive therapy for protecting vital cracked teeth. Nevertheless, multicenter prospective studies with extended follow‐up time and larger sample sizes are needed to confirm the effectiveness of the occlusal veneer for the vital cracked teeth.

## AUTHOR CONTRIBUTIONS

**Mengke Wang:** Conceptualization; data curation; formal analysis; investigation; methodology; project administration; visualization; writing – original draft; writing – review and editing. **Yingying Hong:** Conceptualization; data curation; formal analysis; funding acquisition; investigation; methodology; project administration; resources; software; writing – review and editing. **Xiaomei Hou:** Conceptualization; investigation; methodology; project administration; visualization. **Yinfei Pu:** Conceptualization; data curation; formal analysis; funding acquisition; investigation; methodology; project administration; writing – original draft; writing – review and editing.

## FUNDING INFORMATION

None.

## CONFLICT OF INTEREST STATEMENT

The authors have no conflicts of interest to disclose.

## ETHICS STATEMENT

Institutional review board/ethics committee approval was obtained from the Institutional Review Board of the Peking University School and Hospital of Stomatology (PKUSSIRB‐202272008).

## CONSENT STATEMENT

Written informed consent was obtained from the patient to publish this report in accordance with the journal's patient consent policy.

## INFORMED CONSENT

The patient has consented to the submission of the case.

## PERMISSION TO REPRODUCE MATERIAL FROM OTHER SOURCES

Not included.

## CLINICAL TRIAL REGISTRATION

This study was registered on the webpage of Chinese Clinical Trial Registry and approved by their Institutional Review Board (ChiCTR2200057462, 13/03/2022).

## Data Availability

All authors gave their final approval and agreed to be accountable for all aspects of the work.
